# Pseudokebnorization of leprosy: A unique phenomenon from mpox virus coinfection

**DOI:** 10.1016/j.jdcr.2023.12.021

**Published:** 2024-01-20

**Authors:** Ashley Obi, Aakaash Varma, Lisa Zhou, Israel Kasago, George Niedt, Cula Svidzinski

**Affiliations:** a Department of Dermatology, Icahn School of Medicine at Mount Sinai, New York, New York; b Meharry Medical College, Nashville, Tennessee; c Sadick Dermatology Research Group, New York, New York

**Keywords:** immunosuppression, lepromatous leprosy, leprosy, monkeypox virus, mpox, tuberculoid leprosy

## Introduction

Leprosy is a curable granulomatous infectious disease caused by *Mycobacterium Leprae*, which demonstrates a predilection for skin and nerves.^1^ With approximately 200,000 leprosy cases reported worldwide, leprosy is a reemerging threat. In 2018, there were 185 new cases diagnosed in the United States, with 75% of cases seen in immigrants.^2^ In southern United States, two-thirds of the acquired cases were due to endemic armadillo-derived strains of *M leprae*.^3^ The diagnosis of leprosy is challenging due to its protracted onset, and wide spectrum of clinical manifestations.

Viral infections can give rise to unique immunologic phenomena, which may complicate the clinical picture. From immune reconstitution inflammatory syndrome seen in HIV infection to epitope spreading seen in severe acute respiratory syndrome coronavirus 2 infection, evidence linking viral infection to immune dysregulation continues to build. In 2022, the reemergence of monkeypox (mpox) virus infection attracted worldwide attention with unusual reports of human-to-human and community transmission and a somewhat different clinical presentation than prior outbreaks. mpox virus infection has demonstrated severe impairment in cellular immunity, characterized by a decreased CD4 cell count and impaired natural killer cell function.^4^ Profound immunosuppression may facilitate infection or unmask subclinical infection due to lack of a host immune response. Herein, we describe a unique case of mpox virus infection unmasking leprosy.

## Case report

A 38-year-old Hispanic woman with seronegative biopsy-proven small vessel neuritis, vasculitis, and positive antiphospholipid (APL) antibodies, presented to dermatology with a worsening painful and pruritic papulovesicular eruption of 2 months duration. Five years prior, she immigrated to Texas from the Dominican Republic. She recalled a short-lived cutaneous eruption years ago which included anesthetic hypopigmented patches. About 6 months before she presented to dermatology, she developed progressive neuropathy manifesting as numbness, tingling, and severe disabling pain with edema on bilateral lower extremities. In Texas, a sural nerve biopsy was consistent with inflammatory vasculitic neuropathy. Gomori trichome, Congo red, crystal violet, and toluidine blue stains were negative for amyloid deposition. Treatment included prednisone and mycophenolate mofetil with good response. Two months later, she moved to New York where she established care with rheumatology. Rheumatology initiated prednisone 20 mg daily with plan for maintenance with rituximab for her vasculitis, neuritis, and APL antibodies. Two days after initiating prednisone, she presented to the emergency department with a painful and pruritic papulovesicular eruption on her face, trunk, and bilateral upper and lower extremities (Supplementary Fig 1, available via Mendeley at https://data.mendeley.com/datasets/vwpt8k9hvw/1). She was afebrile and vital signs were stable but extensive review of systems was notable for arthralgias. mpox polymerase chain reaction testing of lesions on the patient’s arm was positive. The patient was discharged with isolation precautions and appropriate quarantine recommendations. One month later, rheumatology initiated rituximab 1000-mg intravenousinfusion. She received a total of 2 doses, each infusion 2 weeks apart. She was also started on trimethoprim/sulfamethoxazole 800 to 160 mg orally 3 times a week as prophylaxis against pneumocystis.

**Fig 1:**
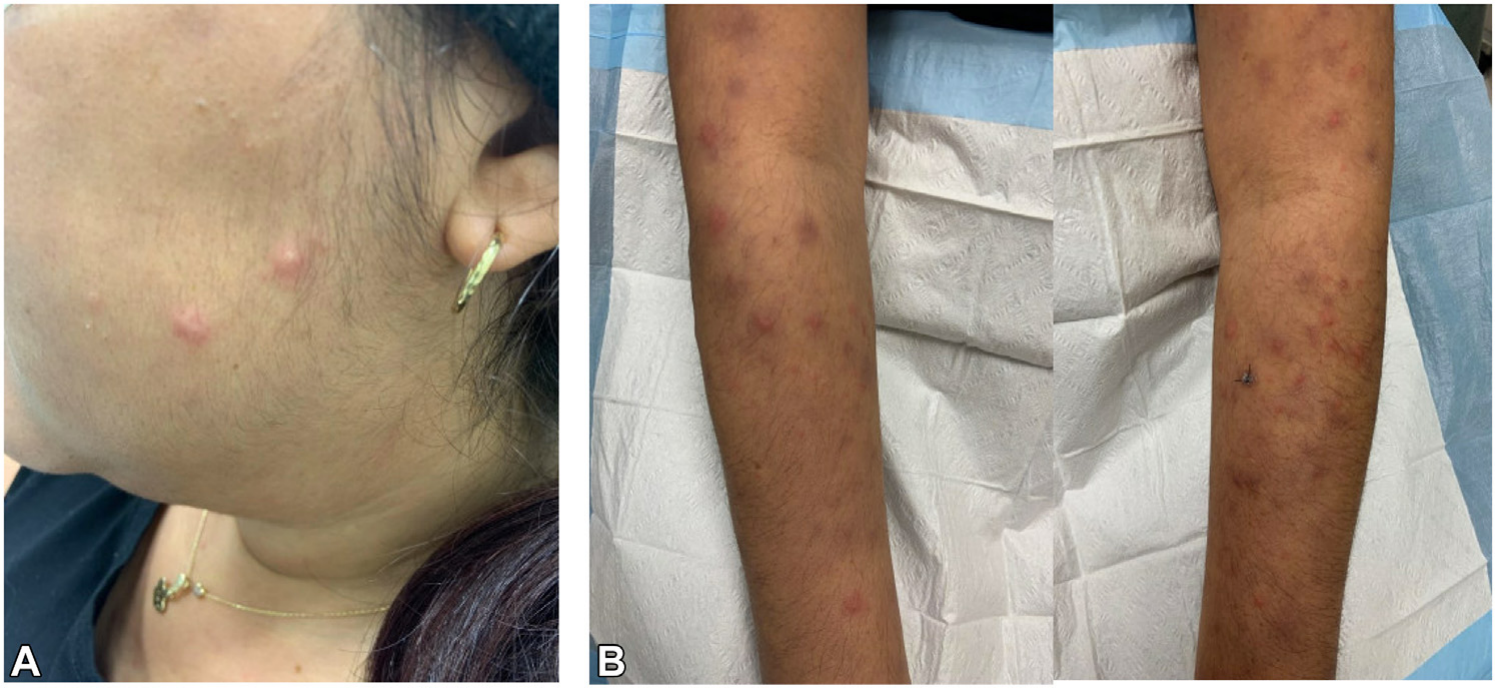
Clinical photographs of a 38-year-old female with multiple *pink*, tender dome-shaped indurated papulovesicles scattered on the face (**A**) and arms (**B**) in the emergency room.

**Fig 2:**
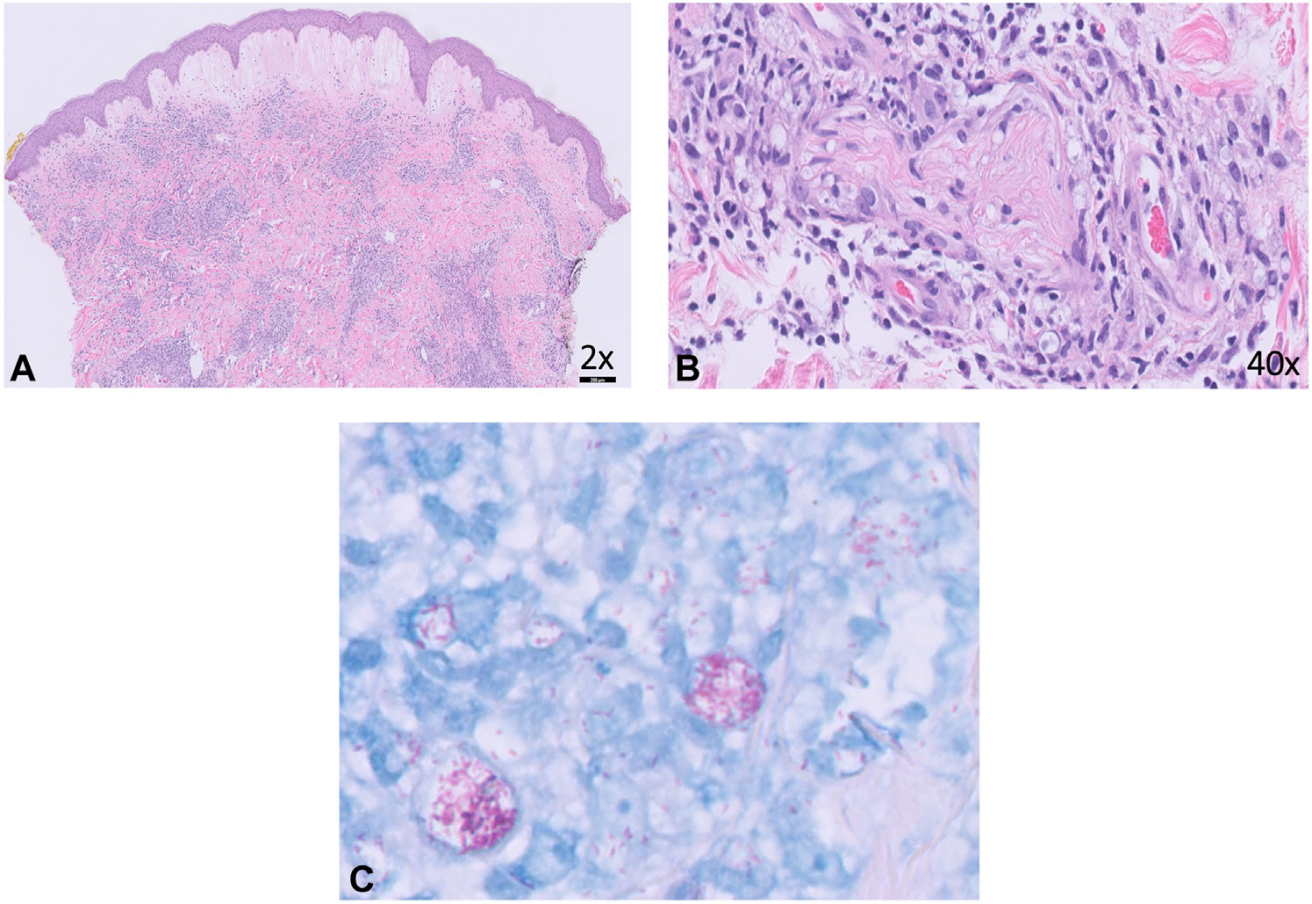
Photomicrograph of hematoxylin and eosin stain demonstrating marked subepidermal edema with superficial and deep perivascular dermatitis (**A**). Hematoxylin and eosin showing epithelioid granulomas and globi surrounding a nerve (**B**). Fite stain demonstrating innumerable AFB within foamy macrophages (**C**).

One month after her last rituximab infusion, she presented to dermatology with persistent painful and pruritic papulovesicular lesions. Extensive review of systems was unremarkable. She denied any recent travel or sick contacts. Her past medical history was notable for migraines. Her medications included aspirin, pantoprazole, pregabalin, sumatriptan, trimethoprim/sulfamethoxazole, and prednisone daily. She was afebrile with stable vital signs. Full body skin examination revealed multiple pink, tender, indurated, dome-shaped papulovesicles with surrounding rim of erythema scattered on the face, trunk, arms, and lower extremities at sites of prior mpox infection (Fig 1). Complete blood count demonstrated leukocytosis (16.8 K/uL) (reference range: 4.5-11 K/uL), neutrophilia (85%) (reference range: 40%-78%), and lymphocytopenia (9.9%) (reference range: 15%-50%) while comprehensive metabolic panel was unremarkable. A punch biopsy demonstrated marked subepidermal edema with superficial and deep perivascular dermatitis composed of epithelioid granulomas with foamy macrophages and globi, which involved few nerves (Fig 2, *A* and *B*). Acid-Fast Bacilli and Fite stains were positive (Fig 2, *C*). Tissue cultures were negative. Polymerase chain reaction isolated *M leprae*. The patient was managed with dapsone 100 mg, clofazimine 100 mg, thalidomide 100 mg, and prednisone 10 mg daily, at a specialized leprosy clinic. Rheumatology held rutiximab.

## Discussion

This is an extremely complicated case. It is unclear whether her autoimmune activation (APL vasculitis/neuritis) preceded the onset of leprosy or is a result of *M Leprae* infection. It is possible that her *M Leprae* infection acted as an inducer of her APL vasculitis/neuritis, given report of anesthetic hypopigmented patches before the onset of her neuropathy. There is a high prevalence of APL antibodies in leprosy and leprosy-related APL symptoms resemble those found in patients with APLsyndrome.^5^ Her recalcitrance to aggressive management of her APL vasculitis/neuritis is likely due to lack of treatment of her chronic indolent paucibacillary/tuberculoid leprosy. Aggressive immunosuppression likely led to a gradual increase of her *Mycobacterium* burden, despite providing initial symptomatic relief of her neuropathy. Interestingly, her mpox coinfection may have further exacerbated her immunosuppression and mediated pseudokoebnerization^6^ of her leprosy at prior mpox lesions/sites. Clinically, this was evident as her leprosy lesions mimicked her prior mpox lesions seen 1 month prior. Multibacillary leprosy lesions vary from papulonecrotic to vesicular. Though leprosy lesions may be vesicular in nature, it was felt that the dome-shaped to umbilicated nature of the patient’s presentation was due to concurrent mpox coinfection. Histologically, while her biopsy displayed features consistent with leprosy, there was also a presence of marked subepidermal edema. This finding, while nonspecific, is not classically seen in leprosy and is a common feature of mpox infection.^7^ Lastly, it is equally plausible that her mpox infection triggered a lepra reaction, an inflammatory reaction seen in leprosy due to fluctuations in host immunity. Although not previously observed in mpox infection, lepra reactions have been reported in multiple studies in patients with leprosy following vaccination against smallpox. It is possible that impairment of cellular immunity due to mpox infection led to a “downgrading” lepra reaction, with movement toward the lepromatous leprosy pole and development of new skin lesions. This case highlights a highly unusual case of leprosy, with a complex clinical presentation due to mpox infection.

## Conflicts of interest

None disclosed.
